# Unveiling Q fever in Latin America: beyond occupational risks to vulnerable populations and environmental transmission

**DOI:** 10.3389/fpubh.2025.1571557

**Published:** 2025-06-09

**Authors:** Danilo Alves de França

**Affiliations:** Department of Veterinary Hygiene and Public Health, São Paulo State University, Botucatu, São Paulo, Brazil

**Keywords:** *Coxiella burnetii*, emerging infectious, environmental transmission, prisons, urban outbreaks, seroprevalence

Q fever is a globally neglected disease that has been reported on all continents. Its non-specific clinical presentation in both humans and animals, combined with its complex and dispersed epidemiology, makes clinical suspicion and diagnosis particularly challenging (1). The etiological agent, *Coxiella burnetii*, has many possible domestic and wild hosts, multiple transmission routes, and the ability to survive for years in the environment (2). Its main transmission route is airborne, and a single bacterial cell is capable of infecting both humans and entire herds, which underscores the difficulty in predicting, controlling, and mitigating the impact of this bacterium (3).

Outbreaks of the disease in the Netherlands and Australia served as a global wake-up call, prompting investigations in Latin America (2). Here, cattle, sheep, and goat production are economic stays, pushing several countries to investigate the occurrence of the disease in their territories. Half of the studies were published in the last 4 years, demonstrating recent progress in research across the continent. However, data remain sparse and incomplete, and countries such as Belize, Costa Rica, Guatemala, Guyana, Honduras, and Suriname have yet to report any cases or evidence of bacterial circulation (4).

Rural workers and veterinarians who handle placentas and birth fluids have shown high infection rates in Latin America, ranging from 1.7 to 61%, based on studies conducted in countries such as Brazil (1.7–29%), Colombia (23.6–61%), Ecuador (43%), and Trinidad and Tobago (4.4–4.6%) (1, 4–7). In ruminants, infection has been widely documented, with prevalences ranging from 0.22% to 60.6%, based on studies conducted in countries such as Argentina (0.22–7%), Brazil (1–55%), Chile (1.4–2%), Colombia (0.46–27%), Costa Rica (1.8–7.7%), Ecuador (14.5–53%), El Salvador (26%), French Guiana (1.7–14%), Guatemala (9%), Mexico (10–28%), Paraguay (45%), Trinidad and Tobago (4–9.4%), Uruguay (10–11.5%), and Venezuela (60.6%). These numbers have been associated with abortions and reduced productivity, especially in cattle, sheep, and goats, reflecting the significant impact of the disease on the regional economy (4, 8). Febrile and pneumonic outbreaks have been reported in slaughterhouses in Barbosa (21°16′00″S, 49°56′57″W), Brazil, and Entre Ríos (32°02′51.72″S, 60°16′51.60″W), Argentina, highlighting the occupational risk and clinical complications of Q fever in these environments. In addition, patients with endocarditis from Brazil were also described (1).

Although this is a reality, Q fever seems to transcend its occupational character and characteristic febrile symptoms, and Latin America has played a significant role in advancing its understanding. Acute Q fever was diagnosed via indirect immunofluorescence assay (IFA) in 21% (129/604) of suspected dengue patients in the state of São Paulo, Brazil, living in large cities far from animal farms (9). These data suggest the possibility of other sources of infection or a much wider dispersal of the spores by wind. Studies indicate that dogs and cats do not play a significant role in this prevalence; however, wildlife has been increasingly associated. Several reports describe newly infected wild animals and an unusual prevalence among workers in forest parks and zoos (10). In the United Kingdom, an outbreak was reported in workers at a cardboard factory, with infection occurring from handling contaminated straw boards, also suggesting that environmental contamination is enough for the disease to occur, without the need for active animals (11). The incidence of Q fever within prisons in French Guiana and Brazil has also raised questions about its epidemiology, as these populations had no contact with animals and were located far from livestock production areas (12, 13). Due to its spore-like resilience, *C. burnetii* can remain infectious for over 40 months, even under unfavorable environmental conditions, suggesting that contamination within or around the prison facilities may have occurred long before the incarceration of the diagnosed individuals. The cited studies highlight that wild animals, dogs, or even rats may serve as potential reservoirs within the prison environment. Airborne transmission is also plausible, given that the prisons are exposed to winds coming from the surrounding tropical rainforests. Another possibility is transmission through animal-derived foods (such as unpasteurized milk and dairy products) included in the inmates' diet. On the other hand, it cannot be ruled out that prisoners were already in the incubation phase of the disease upon entering the prison system; however, this hypothesis is unlikely since these were not isolated cases. Venereal human-to-human transmission also remains poorly understood in the epidemiology of Q fever and should be considered, especially in female prisons (13, 14).

Vulnerable populations are more susceptible to infections, mainly due to a lack of basic sanitation and inadequate hygiene practices. Although Q fever is an airborne disease, significantly higher prevalence rates have been reported among these populations in Latin America. For example, Indigenous communities in Colombia, homeless populations, and Afro-descendant communities (quilombolas) in Brazil have shown Q fever prevalences of 35% (53/150), 23% (44/200), and 15% (30/203), respectively, representing the highest human infection rates ever reported on the continent (15). It was in a prison population in French Guiana that the world's highest incidence of Q fever was reported (12).

In Brazil, patients with thrombocytosis and arthritis from Rio de Janeiro were infected with *C. burnetii*, demonstrating its clinical breadth beyond pneumonia and endocarditis already known (16). *C. burnetii* infection has also been found in injecting drug users, military firefighters during cadet training, and police officers and their working dogs in Brazil, suggesting new sources of exposure (17).

Although urbanization, tourism, and human expansion into natural areas may theoretically increase exposure to *C. burnetii*, current data on wild reservoirs and environmental occurrence in Latin America are limited (10). As such, the association between these anthropogenic changes and the observed infection rates remains speculative. This hypothesis warrants further investigation, particularly through studies focused on the interface between wildlife, environmental changes, and human exposure to Q fever. Similar associations have been described for other zoonotic pathogens under environmental pressure (18), and exploring this possibility for *C. burnetii* could provide valuable insights.

In order to integrate epidemiological findings and support the interpretative nature of this opinion article, we present Figure 1, which visually compares Q fever seroprevalence across different population groups in Latin America.

**Figure 1:**
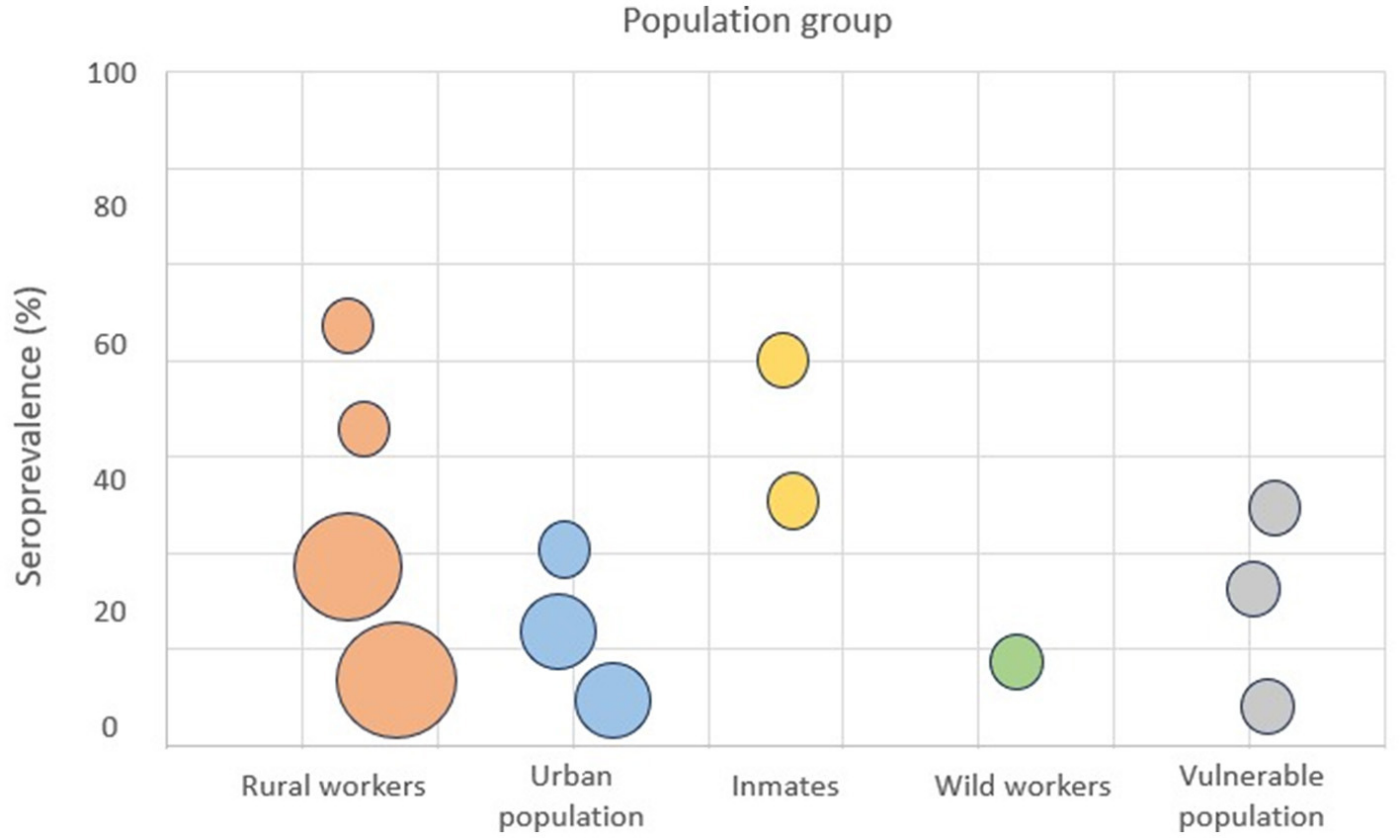
Bubble chart shows the seroprevalence (%) of *Coxiella burnetii* in different population groups in Latin America. X-axis: population groups; Y-axis: seroprevalence (%). Bubble size reflects the sample size or number of studies included in each group. Different colors represent specific population categories (e.g., rural workers, prisoners, indigenous communities, and urban residents).

Notably, high rates are observed not only among rural workers but also in urban, incarcerated, and other vulnerable populations. This challenges the traditional assumption that Q fever is restricted to rural or occupational settings and supports the hypothesis of environmental or foodborne exposure pathways in addition to classical zoonotic routes.

It is crucial to implement specific strategies to advance the understanding of Q fever in Latin America. This includes increasing systematic sampling and testing in human and animal populations, especially in high-risk areas such as rural and peri-urban zones. Complete genomic sequencing and genotyping of positive samples should be prioritized to enable phylogenetic analyses that can elucidate transmission routes and infection sources. These analyses allow the identification of genetic similarities among strains isolated from different hosts and locations, helping to determine whether cases are linked through direct transmission, common environmental exposure, or independent introductions. Furthermore, they can reveal patterns of geographic spread and the emergence of specific genotypes associated with higher virulence or adaptability to certain hosts. This information is essential to understand local epidemiological dynamics and to design more effective control strategies. Additionally, risk mapping using predictive variables such as land use, climate data, population density, and socioeconomic factors can help identify areas of greater vulnerability and guide targeted interventions. The integration of these approaches with strengthened epidemiological surveillance systems can provide valuable insights for the prevention and control of Q fever, reducing its impact on public health and the regional economy.

Finally, we believe that Q fever exists far beyond ruminants as sources of infection and clinical signs that are already known. It is essential to redouble efforts to unravel this disease in all its complexities and to suspect its occurrence in unexpected clinical and epidemiological contexts. Latin American researchers have played a key role in advancing knowledge about Q fever in recent years. However, expanding these findings represents a challenge for health organizations and regional governments. It is necessary not only to encourage new research but also to train local health professionals to recognize the disease and to invest in the development and dissemination of accessible diagnostic tools. In addition, we recommend prioritizing One Health collaborations to integrate wildlife and environmental surveillance with human and veterinary health monitoring and to establish regional reporting systems for Q fever that support data sharing and early outbreak detection.

## References

[1] França DA Mioni MSR Fernandes J Lemos ERS Duré AÍL Silva MVF. Overview of Q fever in Brazil: an underestimated zoonosis. Rev Inst Med Trop Sao Paulo. (2023) 65:e39. 10.1590/s1678-9946202365039 37377322 PMC10313319

[2] Eldin C Mélenotte C Mediannikov O Ghigo E Million M Edouard S. From Q fever to *Coxiella burnetii* infection: a paradigm change. Clin Microbiol Rev. (2017) 30:115–90. 10.1128/CMR.00045-16 27856520 PMC5217791

[3] Bailly S Hozé N Bisser S Zhu-Soubise A Fritzell C Fernandes-Pellerin S. Transmission dynamics of Q fever in French Guiana: a population-based cross-sectional study. Lancet Reg Health Am. (2022) 16:100385. 10.1016/j.lana.2022.100385 36777152 PMC9903881

[4] Epelboin L Mioni MSR Couesnon A Saout M Guilloton E Omar S. *Coxiella burnetii* infection in livestock, pets, wildlife, and ticks in Latin America: a systematic review. Curr Trop Med Rep. (2023) 10:344–58. 10.1007/s40475-023-00288-7

[5] Contreras V Máttar S González M Álvarez J Oteo JA. *Coxiella burnetii* in bulk tank milk and antibodies in farm workers at Montería, Colombia. Rev Colomb Cienc Pecua. (2015) 28:181. 10.17533/udea.rccp.v28n2a0739083843

[6] Echeverría G Reyna-Bello A Minda-Aluisa E Celi-Erazo M Olmedo L García HA. Serological evidence of *Coxiella burnetii* infection in cattle and farm workers: is Q fever an underreported zoonotic disease in Ecuador? Infect Drug Resist. (2019) 12:701–6. 10.2147/IDR.S195940 31114259 PMC6489620

[7] Adesiyun A Dookeran S Stewart-Johnson A Rahaman S Bissessar S. Frequency of seropositivity for *Coxiella burnetii* immunoglobulins in livestock and abattoir workers in Trinidad. New Microbiol. (2011) 34:219–24. 21617835

[8] França DA Silva FPD Zanini DDS Iglesias L Portillo L Cortez H. *Coxiella burnetii* seroprevalence in sheep herd from Paraguay: first evidence of bacterial circulation in the country. One Health. (2023) 18:100660. 10.1016/j.onehlt.2023.100660 38179312 PMC10765107

[9] França DA Mioni MSR Fornazari F Duré AÍL Silva MVF Possebon FS. Seropositivity for *Coxiella burnetii* in suspected patients with dengue in São Paulo state, Brazil. PLoS Negl Trop Dis. (2022) 16:e0010392. 10.1371/journal.pntd.0010392 35536865 PMC9122222

[10] França DA Kmetiuk LB Rodrigues OJD Panazzolo GAK Morikawa VM de Lima Duré AÍ. *Coxiella burnetii* (Q fever) exposure in wildlife professionals. Front Public Health. (2024) 12:1466981. 10.3389/fpubh.2024.1466981 39606082 PMC11599223

[11] Woerden HC Mason BW Nehaul LK Smith R Salmon RL Healy B. Q fever outbreak in industrial setting. Emerg Infect Dis. (2004) 10:1282–9. 10.3201/eid1007.030536 15324550 PMC3323322

[12] Bonifay T Beillard E Daniel M Schiemsky V Vierendeels E Demar M. High incidence of acute Q fever among incarcerated people in Cayenne, French Guiana. Rev Inst Med Trop Sao Paulo. (2022) 64:e42. 10.1590/s1678-9946202264042 35703611 PMC9190515

[13] França DA Kmetiuk LB Pinto GLB Silito IS Kosloski J Duré AÍL. One health behind bars: Seroincidence of *Coxiella burnetii* in women inmates, correctional officers, and feral cats. One Health. (2025) 20:101032. 10.1016/j.onehlt.2025.101032 PMC12235731 40630112

[14] Milazzo A Hall R Storm PA Harris RJ Winslow W Marmion BP. Sexually transmitted Q fever. Clin Infect Dis. (2001) 33:399–402. 10.1086/321878 11438911

[15] Oakley R Dreyfus A Concha G Poppert S Plag M Meile C. Seroprevalence of *Coxiella burnetii* in an indigenous population from the Sierra Nevada De Santa Marta, Colombia. Am J Trop Med Hyg. (2023) 110:155–8. 10.4269/ajtmh.23-0252 37983923 PMC10793008

[16] Rozental T Mascarenhas LF Rozenbaum R Gomes R Mattos GS Magno CC. Coxiella burnetii, the agent of Q fever in Brazil: its hidden role in seronegative arthritis and the importance of molecular diagnosis based on the repetitive element IS1111 associated with the transposase gene. Mem Inst Oswaldo Cruz. (2012) 107:695–7. 10.1590/S0074-02762012000500021 22850965

[17] França DA da Silva JS Rodrigues NJL Duré AÍL Farinhas JH Kmetiuk LB. Serosurvey of *Coxiella burnetii* in police officers and working dogs in Brazil: case report and one health implications. Trop Med Infect Dis. (2024) 9:78. 10.3390/tropicalmed9040078 38668539 PMC11054645

[18] Jones KE Patel NG Levy MA Storeygard A Balk D Gittleman JL. Global trends in emerging infectious diseases. Nature. (2008) 451:990–3. 10.1038/nature06536 18288193 PMC5960580

